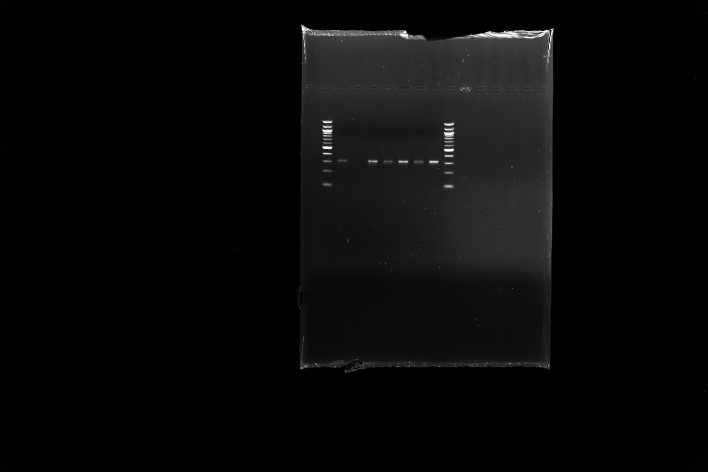# First report of contagious caprine pleuropneumonia (CCPP) in Bangladeshi goats: Seroprevalence, risk factors and molecular detection from lung samples

**DOI:** 10.1016/j.heliyon.2024.e40507

**Published:** 2024-11-19

**Authors:** Md Habibur Rahman, Md Shahin Alam, Md Zulfekar Ali, Md Nurul Haque, Sonia Akther, Sadek Ahmed

**Affiliations:** a Animal Health Research Division, Bangladesh Livestock Research Institute, Savar, Dhaka, 1341, Bangladesh; b SAARC Regional Leading Diagnostic Laboratory for PPR, Bangladesh Livestock Research Institute, Savar, Dhaka, 1341, Bangladesh; c Goat Production Research Division, Bangladesh Livestock Research Institute, Savar, Dhaka, 1341, Bangladesh; d Black Bengal Goat Conservation and Development Research Project, Bangladesh Livestock Research Institute, Savar, Dhaka, 1341, Bangladesh

**Keywords:** Bangladesh, Contagious caprine pleuropneumonia (CCPP), Goats, Molecular detection, *Mycoplasma capricolum* subsp. *capripneumoniae* (Mccp), Seroprevalence

## Abstract

**Background and objective:**

Contagious caprine pleuropneumonia (CCPP) is a highly contagious mycoplasmal respiratory disease primarily affecting goats and sheep caused by *Mycoplasma capricolum* subsp. *capripneumoniae* (Mccp). So far, there is no available information on either the serological or molecular identification of Mccp in Bangladesh. Hence, the objective of this study was to determine the seroprevalence of CCPP and associated risk factors in goats of Bangladesh, as well as molecular identification of the causative agent (Mccp) in this country.

**Materials and methods:**

From July 2022 to June 2023, 402 goat serum samples were randomly collected to determine seroprevalence, and 90 clinically suspected lung samples were collected for molecular confirmation of CCPP. Risk factors were evaluated by interviewing goat owners using a predesigned questionnaire. A commercially available cELISA kit was used to screen blood serum for anti-CCPP antibodies and PCR for Mccp detection. The 16S rRNA gene specific to *Mycoplasma mycoides* cluster (Mmc), and the Mccp-specific gene of *Mycoplasma capricolum* subsp. *capripneumoniae* (Mccp) were amplified through PCR. Potential risk factors were identified through a univariate logistic regression followed by a multivariate logistic regression model.

**Results:**

Out of 402 samples, 29 were tested positive for CCPP, indicating an overall seroprevalence of 7.21 % (95 % CI: 1.90–12.53). The PCR result showed that 26.67 % of the samples were positive for CCPP. The associated risk factors for the disease were animal age (>18 months; OR: 2.14, 95 % CI: 0.92–4.98), sex (Female; OR: 5.80, 95 % CI: 1.70–19.69), flock size (Large; OR: 6.28, 95 % CI: 1.17–35.74), and body condition scores (Poor; OR: 5.58, 95 % CI: 1.36–22.92).

**Conclusion:**

This study confirms the existence of CCPP in Bangladeshi goats for the first time using both serological and molecular methods (PCR).

## 1 Introduction

Goats play a vital role in ensuring adequate nutrition, food security, and economic independence for their owners, particularly landless small-scale farmers in several lower-income countries [1,2]. Goats are an integral part of the livestock sector in Bangladesh, with an overall 26.95 million population, constituting 47.15 % of the country's entire ruminant population [3]. Goat farming is gaining popularity to meet the increasing demand for animal protein in Bangladesh. However, the high humidity and hot weather of Bangladesh seem to facilitate the emergence of various diseases, thereby decreasing animal productivity and escalating veterinary expenses [4,5]. Respiratory diseases in goats and sheep are responsible for significant economic losses around the world [6,7] and also cause high morbidity and mortality in Bangladesh [8]. Depending on the cause, pathogenic respiratory diseases of sheep and goats account for 5.6 percent of all the small ruminant diseases responsible for nearly 50 % of mortality [6,9]. However, goat production is hampered by several infectious respiratory diseases, and among them, the most serious is contagious caprine pleuropneumonia [10,11].

Contagious caprine pleuropneumonia (CCPP) is a deadly mycoplasmal respiratory disease of goats that causes substantial financial damage, as listed by the World Organization for Animal Health [6,12]. CCPP is a common transboundary livestock disease that has greatly hampered goat production and productivity due to its rapid spread [13,14]. The causal organism for CCPP is *Mycoplasma capricolum* subsp. *Capripneumoniae,* generally known as Mccp and formerly as Mycoplasma F38 a *Mycoplasma mycoides* cluster (Mmc) member [[14], [15], [16], [17]]. This is one of the tiniest fastidious bacteria (300 nm) without a cell wall but possessing a triple-layer surface [18]. In Algeria, about 1873, the first clinical case of CCPP was recorded [19]. After 103 years, the actual CCPP-causal agent, Mccp was finally identified and described in 1976 [20,21]. However, Mccp is currently identified in 13 countries and has been suspected clinically in over 40 countries [22]. Anorexia, high fever (41^0^C–43 °C), dyspnea, polypnea, coughing, grunting, failure to move, rigid neck and persistent salivation along with nasal discharges are frequently seen as clinical signs of CCPP [[23], [24], [25], [26]]. In addition, abortion and a significant rate of mortality have also been documented [18,27]. During the post-mortem, the animal showed fibrinous pleuropneumonia accompanied by pleurisy, extensive lung hepatization, straw-colored fluid in the pleura, froth in the trachea, and enlarged respiratory lymph nodes [10,23]. Both domestic and wild ruminants are susceptible to CCPP, which causes 100 % morbidity in non-endemic regions and 30–40 % in the endemic regions, with a mortality rate reaching 80–100 % [24,28]. It is more common in domesticated goats but also seen in sheep reared with goats [15,23]. All ages and sexes are susceptible, while young goats are more affected than adults [15]. The primary mode of transmission is through the inhalation of contaminated droplets. Airborne transmission may result in dissemination over long distances, and a 50-m distant transmission is recorded [23,29]. Low immunological state, present viral infection, large herd size, sudden changes in the climate, old age, and distress may encourage CCPP development [10,30]. CCPP does not exhibit zoonotic characteristics and no evidence of the potential for human infection by Mccp [31].

Detection of Mccp infection poses challenges due to the variability of the clinical signs or symptoms [7,22]. Normally, this organism is identified using polymerase chain reaction (PCR) and serological techniques, which may be readily employed on clinically suspected samples [32]. Serological assays, such as Enzyme-Linked Immunosorbent Assay (ELISA) and latex agglutination test (LAT), are significant tools in the detection of CCPP in animals [23]. At the same time, PCR is widely regarded as a highly specific and precise method for diagnosing Mccp. Although this technique is time-consuming, high cost, and exhibits limited efficacy in detecting Mccp in animals undergoing antibiotic treatment. In contrast, ELISA can detect Mccp infection in both antibiotic-treated and cured animals [22]. Thus, we used both serological and molecular methods to determine goat CCPP in the study areas.

CCPP is emerging as a severe concern for countries that have never had this highly infectious disease or are in danger due to frequent trade interactions with affected countries or their neighbors [23]. CCPP in India[[33], [34], [35], [36]], Nepal [16], and Pakistan [13,18,26] may raise the chance of an outbreak in Bangladesh. The wide borders between Bangladesh and neighboring countries, inadequate disease monitoring, and deficient quarantine facilities contribute to an increased risk of CCPP in Bangladesh. Frequent livestock movement between neighboring countries of Bangladesh could cause CCPP in Bangladeshi small ruminants. However, there is no available serological or molecular evidence about the infection of CCPP in Bangladesh. So, the objectives of this study were to determine the seroprevalence of CCPP and related risk factors in Bangladeshi goats and also identify the causal agent molecularly for the first time in Bangladesh. The analysis of seroprevalence gives valuable scientific information about disease occurrence in certain regions, including risk factors like breed, age, sex, and seasonal disease outbreaks and molecular identification gives the knowledge about the causal agent. Therefore, the current study gives baseline information for developing possible approaches for diagnosing, preventing, and controlling this infectious disease in the future in Bangladesh.

**Fig. 1:**
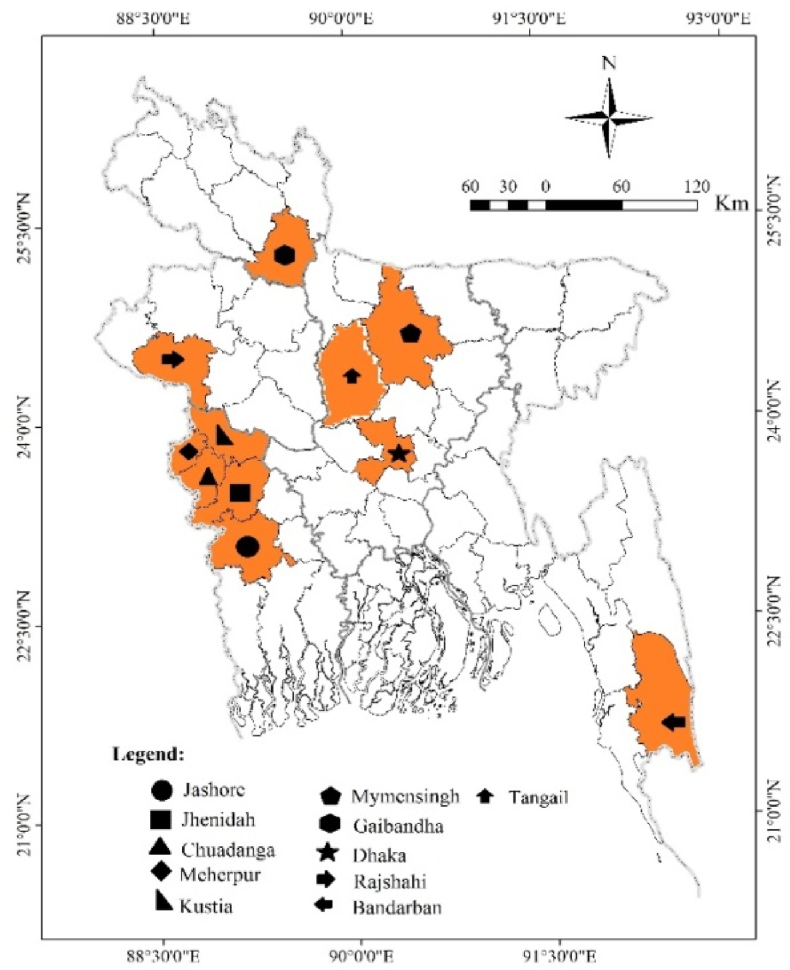
The sampling sites are shown on the map of Bangladesh.

## 2 Materials and methods

### 2.1 Study location, period and animals

The study was designed from July 2022 to June 2023 to investigate both the seroprevalence and molecular identification of contagious caprine pleuropneumonia (CCPP) in goats in 11 districts of Bangladesh (Fig. 1). Total 402 blood samples were collected randomly from goats residing in study areas, namely Dhaka (n = 42), Jhenidah (n = 31), Jashore (n = 44), Mymensingh (n = 32), Rajshahi (n = 38), Gaibandha (n = 35), Kustia (n = 35), Meherpur (n = 43), Tangail (n = 30), Bandarban (n = 34), and Chuadanga (n = 38). Goat owners were interviewed directly to determine risk factors. The study involved the collection of samples from male and female goats of Jamunapari (JP), Black Bengal goat (BBG), and crossbreeds among three distinct age groups: less than 6 months (kid), 6–18 months (growing), and above 18 months (adult). Additionally, ninety (90) lung samples were collected from deceased goats suspected of having CCPP for molecular identification. In the study area, goats were raised using free-range and semi-intensive system. All goats included in the study were vaccinated against Peste des Petits Ruminants (PPR).

### 2.2 Calculating sample size

There was no prior research on CCPP prevalence in Bangladesh, so the Thrusfield (2018) [37] formula was applied to compute the sample size with 50 % expected prevalence in 95 % confidence interval and 5 % desired absolute precision.

n = 1.96^2^P_exp_ (1-P_exp_)/d^2^

where,

n = required sample size, P_exp_ = expected prevalence and d = desired absolute precision (5 %)

Hence, this study needed 384 goats; however, 402 goats were sampled for maximum precision.

### 2.3 Data collection

Interviews with goat owners or representatives using structured questionnaires to gather detailed information on goat-rearing practices. Sex, age, housing, breed, flock size, rearing system, and body condition score were gathered during the interview.

### 2.4 Sample collection

**Fig. 2:**
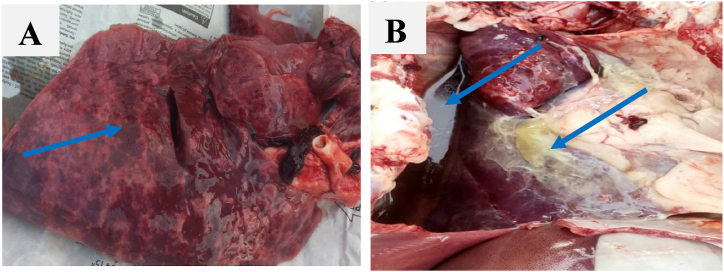
CCPP suspected lung samples for molecular confirmation. A) Lung showing congestion and red hepatization B) Lung covered with yellow coat of fibrin and straw-colored pleural fluids. (For interpretation of the references to color in this figure legend, the reader is referred to the Web version of this article.)

Five (5) ml of blood were collected from the jugular vein of each goat using an aseptic syringe. The collected blood samples were subsequently sent to the Small Ruminants Research Laboratory of the Bangladesh Livestock Research Institute by ensuring cold chain for further study. Blood samples were centrifuged at 2000 rpm for 15 min to extract serum from blood samples, which were kept at −20 °C in a 2 ml pre-labelled Eppendorf tube until use. The lung tissues were collected from suspected CCPP goats during necropsy to perform molecular detection of Mccp. All suspected lung samples [Fig. 2(A and B)] were collected from the affected and healthy lung tissue aseptically. The lungs exhibiting extensive unilateral hepatization were individually gathered and placed in sterilized zip-locked bags. All samples were brought to the lab in an icebox and maintained at −20 °C in a deep freezer and further tests were done within 72 h.

### 2.5 Serological test

All serum samples were tested with a commercially available ELISA kit (IDEXX-CCPP, Montpellier, France, Lot: SN 21124; Ref: 99–56231) for the presence of anti-CCPP antibodies according to the manufacturer's instructions. The optical density (OD) values were calculated using an ELISA plate reader (Multiskan FC, Thermo Fisher Scientific, USA) at a wavelength of 450 nm. Then, using the obtained OD values from the tested samples, percentage of inhibition (PI) was calculated according to the formula given by the manufacturer (IDEXX-CCPP, Montpellier, France) to find out the positive and negative samples (Supplementary file). Samples exhibiting a PI value above or equal to 55 % were classified as positive, and samples having a PI value below 55 % were classified as negative for CCPP.

### 2.6 Risk factors

The variables evaluated in this study to find out the association with the seroprevalence of CCPP among goats included breed, age, sex, flock size, rearing system, body condition score (BCS), and housing systems. The statistical significance of the results was established when the calculated p-value was below or equal to 0.05 (by using 95 % confidence of interval).

## 3 Molecular detection of *Mycoplasma capricolum* subsp. *capripneumoniae* (Mccp) by PCR

### 3.1 Extraction and quantification of DNA

A commercial DNA extraction kit (PureLink™ Genomic DNA Mini Kit, Invitrogen, Thermo Scientific, USA, Lot: 2417493, Ref: K182001**)** was used for bacterial DNA extraction from lung tissues by following the manufacturer's instructions. The extracted DNA's purity (wavelength 260/280) and concentration were measured using μDrop (Thermo Fisher Scientific, USA) and found between 1.8 and 2.0 and 40–**1**80 μg/ml, respectively (Supplementary file 2).

### 3.2 Polymerase chain reaction (PCR) and amplification

First of all, to confirm the presence of *Mycoplasma mycoides* cluster (Mmc), PCR was conducted using a particular set of primers targeting the 16S rRNA gene, as designed by Hotzel et al. [38] (Table 1). The predicted size of the amplified DNA fragment was 548 base pairs (bp). Subsequently, after confirming the mycoplasma cluster, Mccp-gene-specific primers were designed by Woubit et al. [32] (Table 1) for *Mycoplasma capricolum* subsp. *capripneumoniae* were used, with the predicted size of the amplified DNA fragment was 316 bp, which confirmed the detection of Mccp. PCR was carried out using a Thermal Cycler (Bio-Rad, USA). Details of the primer sets, reaction volume, and PCR condition are summarized in Table 1. Following PCR amplification, 5 μl of the amplified PCR products were subjected to gel electrophoresis on a 1.5 % agarose gel (w/v) by providing 100V and 500 mA current for 30 min and visualized using ethidium bromide. The results were analyzed using GelDoc Go Imaging System (Bio-Rad, USA).

**Table 1:** List of primers, reaction volume, and conditions used in PCR.

Gene	Primer sequence	Reaction volume (25 μl)	PCR Conditions	Amplicon size	References
16S rRNA	F 5′-CGAAAGCGGCTTACTGGCTTGTT-3′ R 5′-TTGAGATTAGCTCCCCTTCACAG-3′	GoTaq green Master mix- 12.5 μl, Forward primer- 1.0 μl, Reverse primer- 1.0 μl, Nuclease free water- 5.5 μl, DNA template- 5 μl	Initial denaturation 95 °C, 4 min Denaturation (35 cycles) 95 °C, 45 s Annealing 55 °C, 1 min Extension 72 °C, 1 min Final extension 72 °C, 5 min	548 bp	[37]
Mccp-spec	F 5′- ATCATTTTTAATCCCTTCAAG -3′ R 5′- TACTATGAGTAATTATAATATATGCAA -3′		Initial denaturation 94 °C, 2 min Denaturation (35 cycles) 94 °C, 30 s Annealing 47 °C, 15 s Extension 72 °C, 15 s Final extension 72 °C, 5 min	316 bp	[31]

### 3.3 Statistical analysis

Microsoft Excel 2016 (Microsoft Corp) was used to edit and clean datasets. The statistical analysis was done with STATA-14.2 (StataCrop, USA). Descriptive statistics were used to compare CCPP seropositivity to goat breed, age, sex, rearing system, flock size, BCS, housing system, and location. Associational analyses were conducted on two distinct levels. The univariate logistic regression analysis was used to examine the relationships between risk factors, which were transformed into categorical variables, and CCPP seropositive outcomes. After that, a multivariable logistic regression model was used for determining potential risk factors. The outcomes for every single predictive variable were presented as odds ratio (OR) along with a 95 % confidence interval (CI). The factors related to seropositivity were determined through a combination of forward selection and backward elimination techniques. At last, factors that exhibited statistically significant p-values (<0.05) were determined to be the primary contributors to the seropositivity of goats in CCPP.

## 4 Results

### 4.1 Seroprevalence of CCPP by cELISA

A total of 402 goats were included in this study to determine the seroprevalence of contagious caprine pleuropneumonia (CCPP) using the cELISA test (Table 2). The findings revealed that the overall seroprevalence of CCPP in goats was 7.21 % (95 % CI: 1.90–12.53). The highest seroprevalence, 11.90 % (95 % CI: 3.98–25.63) was observed in the Dhaka district, while goats from the Gaibandha district showed the lowest seroprevalence at 2.86 % (95 % CI: 0.07–14.91). Notably, no CCPP antibodies were found in goats from Tangail. In addition, statistical analysis showed that there were no significant differences (P < 0.05) in the prevalence of seropositivity to CCPP across the eleven districts (Table 2).

**Table 2:** District-wise seroprevalence of CCPP in goats tested by cELISA.

Sampling sites	No. of sera tested	Positive	Prevalence	95 % CI	OR (95 % CI)	*P*-value
Jashore	43	4	9.30 %	2.59–22.13	3.48 (0.37–32.72)	0.274
Jhenidah	31	1	3.23 %	0.08–16.70	1.13 (0.06–18.91)	0.931
Chuadanga	39	2	7.69 %	1.61–20.87	2.83 (0.28–28.57)	0.377
Meherpur	43	5	11.63 %	3.88–25.08	4.47 (0.49–40.22)	0.181
Kustia	35	3	8.57 %	1.80–23.05	3.18 (0.31–32.24)	0.326
Rajshahi	38	3	7.89 %	1.65–21.37	2.91 (0.28–29.41)	0.365
Mymensingh	32	1	3.13 %	0.07–16.21	1.09 (0.06–18.29)	0.949
Dhaka	42	5	11.90 %	3.98–25.63	4.59 (0.51–41.34)	0.174
Bandarban	34	4	11.76 %	3.30–27.45	4.53 (0.47–42.82)	0.187
Gaibandha	35	1	2.86 %	0.07–14.91	Ref.	–
Tangail	30	0	–	–	–	–
**Total**	**402**	**29**	**7.21 %**	**1.90–12.53**		

∗CI- Confidence Interval; OR- Odds Ratio.

### 4.2 Univariate analysis

The results of a univariable logistic regression analysis examining the risk factors for CCPP are presented in Table 3. When comparing different goat breeds, the cross-breed goats had the highest seroprevalence of CCPP at 9.89 % (95 % CI: 5.96–15.17) compared to Black Bengal goats (5.81 %; 95 % CI: 2.68–10.73), and Jamunapari goats (4.62 %; 95 % CI: 0.96–12.90). However, no statistically significant differences were found between the breeds. A higher rate of seroprevalence was recorded in adult goats (10.12 %, 95 % CI: 6.0–15.70, OR: 1.94) in comparison to both kids (<6 months) and growing goats (6–18 months). A highly significant variation in seropositivity was found between the sexes of goats (p < 0.05), and a higher prevalence was found in female goats (9.47 %, 95 % CI: 6.09–16.86, OR: 2.27). Additionally, a significant variation in seropositivity was observed among goats with varying body condition scores (BCS) (p < 0.05). Specifically, goats with low BCS exhibited a higher prevalence of seropositivity (9.38 %, 95 % CI: 4.37–17.05, OR: 1.4) compared to the other groups. Furthermore, the semi-intensive rearing method showed a higher seroprevalence (8.92 %, 95 % CI: 5.45–13.57, OR: 1.58) than the free-range rearing method. The study found a higher seroprevalence in large flock size (11.31 %, 95 % CI: 6.94–17.09, OR: 2.86) than in small and medium flock size. In terms of housing systems, it was shown that the seropositivity of CCPP was higher in goats housed in slat systems (8.21 %, 95 % CI: 4.85–12.82, OR: 1.25) compared to those housed in floor systems, but this association was not statistically significant.

**Table 3:** Univariable logistic regression analysis of risk factors for contagious caprine pleuropneumonia (CCPP) seropositivity of goats in the study area by cELISA.

Variable	Category	No. of tested (% positive)	95 % CI	OR (95 % CI)	*P*-value
Breed	Jamunapari	65 (4.62)	0.96–12.90	Ref.	
	Black Bengal goat	155 (5.81)	2.68–10.73	1.27 (0.33–4.86)	0.723
	Cross-breed	182 (9.89)	5.96–15.17	2.26 (0.64–7.97)	0.201
Age (month)	Kid (<6)	52 (5.77)	1.20–15.94	1.05 (0.27–3.97)	0.939
	Growing (6–18)	182 (5.49)	2.66–9.87	Ref.	
	Adult (>18)	168 (10.12)	6.0–15.70	1.94 (0.86–4.35)	0.110
Sex	Male	159 (4.40)	1.78–8.86	Ref.	
	Female	243 (9.47)	6.09–16.86	2.27 (0.95–5.42)	0.035
Rearing system	Free	189 (5.82)	2.94–10.17	Ref.	
	Semi Intensive	213 (8.92)	5.45–13.57	1.58 (0.73–3.42)	0.241
Flock size	Small	187 (4.81)	2.22–8.93	1.14 (0.23–5.45)	0.872
	Medium	47 (4.26)	0.51–14.54	Ref.	
	Large	168 (11.31)	6.94–17.09	2.86 (0.64–12.79)	0.167
BCS	Good	117 (6.84)	2.99–13.02	Ref.	
	Medium	189 (6.88)	3.71–11.47	1.006 (0.40–2.50)	0.989
	Poor	96 (9.38)	4.37–17.05	1.4 (0.52–3.80)	0.041
Housing system	Floor	195 (6.67)	3.59–11.13	Ref.	
	Slat	207 (8.21)	4.85–12.82	1.25 (0.59–2.65)	0.556

∗CI- Confidence Interval; OR- Odds Ratio.

### 4.3 Multivariate analysis

In multivariate logistic regression analysis, four variables (age, sex, flock size, and BCS of goats) were predicted as potential risk factors for the seroprevalence of CCPP in goats in the selected study areas. These factors were examined in relation to each other to determine their predictive value. The study's results showed that adult goats over 18 months (OR: 2.14, 95 % CI: 0.92–4.98), female goats (OR: 5.80, 95 % CI: 1.70–19.69), large flock size (OR: 6.28, 95 % CI: 1.17–35.74), and goats with low BCS (OR: 5.58, 95 % CI: 1.36–22.92) were more likely to test positive for CCPP. These findings are summarized in Table 4.

**Table 4:** Results of multivariable logistic regression analysis of potential risk factors associated with contagious caprine pleuropneumonia (CCPP) seropositivity of goats by cELISA.

Variable	Category	Adjusted OR (95 % CI)	*P*-value
Age (month)	Kid (<6)	1.47 (0.36–5.87)	0.582
	Growing (6–18)	Ref.	
	Adult (>18)	2.14 (0.92–4.98)	0.047
Sex	Male	Ref.	
	Female	5.80 (1.70–19.69)	0.005
Flock size	Small	1.37 (0.18–10.14)	0.755
	Medium	Ref.	
	Large	6.28 (1.17–35.74)	0.038
BCS	Good	Ref.	
	Medium	2.39 (0.77–7.36)	0.127
	Poor	5.58 (1.36–22.92)	0.017

∗CI- Confidence Interval; OR- Odds Ratio.

### 4.4 PCR-based molecular confirmation of Mccp

For molecular confirmation, 90 suspected lung samples were subjected to PCR where primers specific to 16S rRNA gene and Mccp-specific-gene were used to detect the *Mycoplasma mycoides* cluster and *Mycoplasma capricolum* subsp*. capripneumoniae*, respectively. The result showed that out of 90 suspected lung samples, 41 were *Mycoplasma mycoides* cluster and 24 were identified as *Mycoplasma capricolum* subsp. *capripneumoniae.* The PCR method revealed a molecular detection rate of Mccp at 26.67 % (n = 90, 95 % CI: 17.32–36.02).

**Fig. 3:**
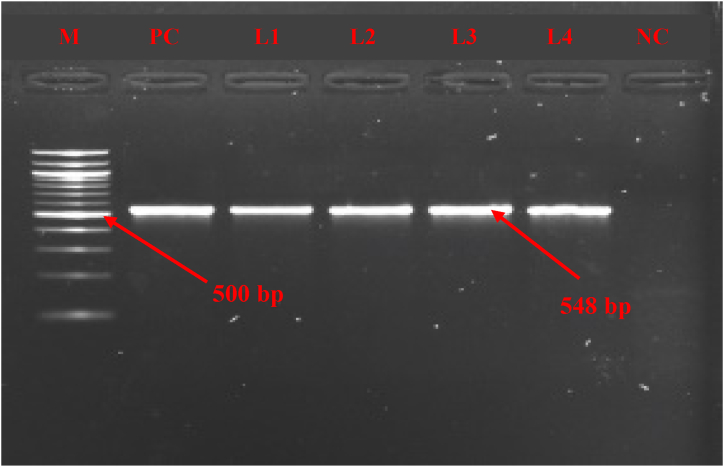
**(**Supplementary file) PCR analysis revealed the presence of *Mycoplasma mycoides* cluster in the tested lung samples, as indicated by an amplicon size of 548 bp. M = 100 bp DNA ladder, samples = L1, L2, L3, L4, PC = positive control and NC = negative control.

**Fig. 4:**
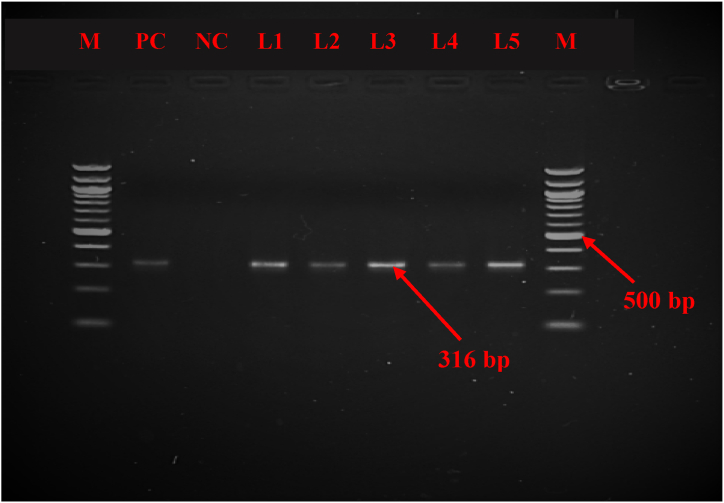
**(**Supplementary file) PCR analysis revealed the presence of *Mycoplasma capricolum* subsp. *capripneunomiae* (Mccp) in the tested lung samples, as indicated by an amplicon size of 316 bp. M = 100 bp DNA ladder, PC = positive control, NC = negative control and samples = L1, L2, L3, L4, L5.

The confirmation of the *Mycoplasma mycoides* cluster depicted in Fig. 3 was done by applying universal primers (548 bp) with the 16S rRNA amplification during PCR. The amplification of the Mccp-specific-gene (316 bp) was performed to identify *Mycoplasma capricolum* subsp. *capripneumoniae,* as depicted in Fig. 4.

## 5 Discussion

Contagious caprine pleuropneumonia holds significant economic importance in goat farming, as it directly impacts production, particularly in rural areas where goat production is increasing, especially in developing countries [11]. This disease is prevalent worldwide, but its incidence is higher in developing and poorly developed regions such as African and Asian countries [39]. CCPP shares similarities with Peste des petits ruminants (PPR) regarding its clinical presentation, signs, symptoms, morbidity, and mortality rate [40]. Consequently, farmers often encounter difficulties in distinguishing between CCPP and PPR. Currently, there is no relevant data regarding the prevalence and molecular confirmation of CCPP infection in goats in Bangladesh. This study is the first instance of conducting epidemiological research and molecular confirmation of contagious caprine pleuropneumonia disease in goats from Bangladesh. In Bangladesh, there has been a notable occurrence of pneumonia among PPR-vaccinated goats currently [41]. According to Rahman et al. [4] and Munsi et al. [5], a significant proportion of PPR-vaccinated goats were found to be affected with pneumonia in Bangladesh, but the etiological agents behind this are still unknown. CCPP currently gets little attention in Bangladesh, which has led to its widespread prevalence being either incorrectly diagnosed or ignored at veterinary clinics and hospitals. Therefore, the findings of the current study give extensive knowledge of the molecular confirmation and serological evidence of CCPP in goats of Bangladesh. Employing cELISA, Bangladeshi goats have a 7.21 % seroprevalence of CCPP. Nearly similar seroprevalence was documented in Borana, Ethiopia 7.1 % [42], western Amhara, Ethiopia 8.5 % [43], and Panjab, Pakistan 8.52 % [26]. The findings of this study indicate a significantly lower prevalence rate compared to other regions/countries in which it is lower than the prevalence rate observed in Kenya (48.6 %) [44], Northern Ethiopia (43.93 %) [45], Southern Ethiopia (27.8 %) [46], Oman (28 %) [47], Sindh, Pakistan (18 %) [13] and Tajikistan (10.1 %) [48]. In contrast, the prevalence rate of this study is higher than Nepal (3.37 %, 4.71 %) [16,49], Pakistan (3.91 %) [18] and Ethiopia (5.1 %, 4.92 %) [10,50]. CCPP prevalence varies by region due to climate, animal management, and housing, as prior research has linked these factors to the transmission of infectious diseases [51,52]. Unrestricted movement of animals, farming systems, ill goat sampling, and agricultural ecology may affect CCPP prevalence rates [30,53]. However, subclinical infection may explain the low seroprevalence. Moreover, it should be noted that the present study cannot be directly compared to previous studies due to inherent differences in sample size, timing and location of sampling, changes in breed disease susceptibility, disparities in husbandry practices, and utilization of different testing methods.

Many risk factors, such as breed, animal age, sex, flock size, and husbandry practices have been identified as contributing to a higher prevalence of CCPP among many countries [13,33,47]. The results of this current study support the concept that there is a higher prevalence of CCPP seropositivity among crossbreed goats, goats aged above 18 months, female goats, and large flock sizes. The present study found a significant relationship between sex and seroprevalence of CCPP, where females are significantly more positive than males. The outcome aligns with the conclusions drawn by Parray et al. [33], Regmi et al. [49], and Fasil et al. [54]. The potential cause of this occurrence can be a decrease in immunity levels because of malnutrition and a lower state of health in female goats. However, the current findings contradict Suryawanshi et al. [36], as they observed a higher prevalence of seropositivity in male goats compared to female goats. The potential factor contributing to the disparity was the low sample size and the higher sample collected from male goats.

In the present study, a significant increase in the seropositivity of CCPP was observed among adult animals compared to the other age groups. The reduced seropositivity observed in young animals may be attributed to humoral immunity [13,39]. Similarly, many researchers found a higher prevalence of CCPP among older goats compared to younger goats [13,33,43,54]. Factors such as adverse weather, long-distance travel, and food deprivation can enhance the susceptibility of animals to diseases. Additionally, the likelihood of an animal encountering many infectious organisms increases as it ages.

The present study found that crossbred goats had a higher seropositivity rate than Black Bengal and Jamunapari goats. However, no statistically significant association was found. The disparity in observed seropositivity may be ascribed to variations in the sample size which is higher sampling from this particular breed. Due to the potential for interspecies transmission of the disease, it is important to incorporate sheep into the existing preventative methods [15,47].

CCPP seroprevalence was influenced by husbandry practices, with a higher prevalence found in semi-intensive farming systems than in free-range systems. Additionally, the findings of this study indicate that the occurrence of CCPP is significantly higher in large flock size (more than 50 goats) compared with small (1–10 goats) and medium-sized flock (11–50 goats). Increased stock density levels, larger flock size, and using semi-intensive rearing systems are associated with a higher prevalence of CCPP [47,50]. In this context, overcrowding and confinement facilitate the proliferation of close interactions and disease transmission among the goat herd.

Furthermore, the current study observed a significant disparity in seropositivity levels among goats exhibiting different body condition scores (BCS). Goats with lower BCS had a higher prevalence than those with moderate and good BCS. This finding is consistent with Adeyemi et al. [55], who claimed that goats having good BCS have lower susceptibility to infectious diseases. However, goats with low BCS are susceptible to many metabolic, bacterial, viral, and parasitic diseases [56].

The definite diagnosis for CCPP involves two methods: isolation of the causal agent, *Mycoplasma capricolum* subsp. *capripneumoniae* from clinical samples or identification of Mccp directly in clinical samples using molecular techniques [27]. Nowadays, several PCR-based techniques have been developed to detect DNA molecules of Mccp in laboratory cultures and directly from clinical samples. These methods have shown remarkable specificity and sensitivity [32]. Using species-specific primers has facilitated the direct application of an updated method to clinical samples, such as lung tissues and pleural fluid [57]. This study used PCR to directly identify and confirm Mccp in clinical samples for the first time in Bangladesh. Ninety (90) lung samples were collected from deceased goats suspected of clinically presenting CCPP, and all the samples underwent PCR to identify the presence of *Mycoplasma capricolum* subsp. *capripneumoniae.* PCR was used to confirm CCPP in 26.67 % (24/90) of the goats, all with CCPP clinical symptoms. Similar studies [7,22,27,[57], [58], [59]] used the same primers and PCR technique to molecularly identify Mccp in lung tissue, although prevalence rates varied. The prevalence rate may vary depending on the nature of the sample, the number of samples, and the testing methods used in those studies. The remaining ones were seen as PCR-negative. This could have happened because different *Mycoplasma* cluster species developed goat diseases with the same signs and symptoms or maybe the presence of another organism like *Pasteurella* spp.

## 6 Conclusion

This study provides the first-ever confirmation of the presence of contagious caprine pleuropneumonia (CCPP) in goats of Bangladesh, supported by both serological evidence and molecular analysis. Age, sex, flock size, and body condition scores significantly affected goat seropositivity. Subsequent investigations will prioritize the isolation of Mccp and following gene sequencing of the detected Mccp. Following the isolation and sequencing of Mccp, it is recommended to conduct preliminary trials aimed at developing a vaccine utilizing locally isolated Mccp strains. However, in the meantime, it is advisable to import a vaccine and immunize goats to reduce the impact of CCPP in goat herds and mitigate the associated economic losses.

## CRediT authorship contribution statement

**Md Habibur Rahman:** Writing – review & editing, Writing – original draft, Software, Methodology, Formal analysis, Data curation, Conceptualization. **Md Shahin Alam:** Writing – review & editing, Supervision, Investigation. **Md Zulfekar Ali:** Writing – review & editing, Software, Formal analysis, Data curation. **Md Nurul Haque:** Writing – review & editing, Methodology, Data curation. **Sonia Akther:** Writing – review & editing, Supervision. **Sadek Ahmed:** Writing – review & editing, Supervision, Funding acquisition.

## Ethical approval

The Animal Experimentation Ethics Committee of Bangladesh Livestock Research Institute approved this research project (Reference no.: AEEC/BLRI00110/2023). During sample collection, all guidelines for animal care were carefully followed.

## Data availability

The datasets used and/or analyzed during the current study are available from the corresponding author on reasonable request.

## Funding

This study was supported by the Black Bengal Goat Conservation and Development Research Project (Grant No. 224289000) of the Ministry of Fisheries and Livestock, Bangladesh.

## Declaration of competing interest

The authors declare that they have no known competing financial interests or personal relationships that could have appeared to influence the work reported in this paper.

## Figures and Tables

**figs1:**
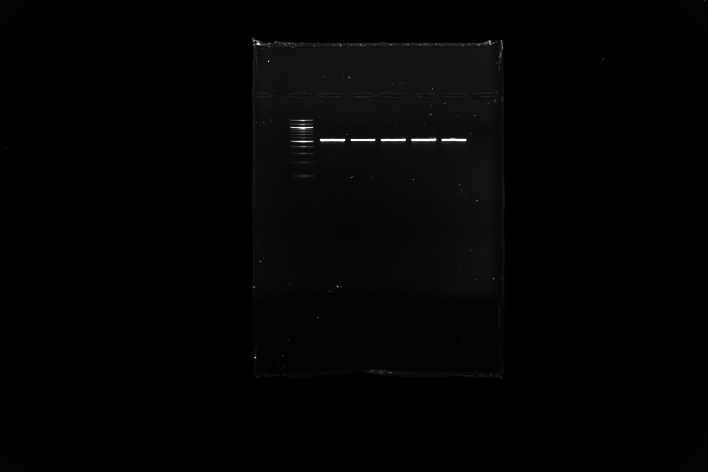


**figs2:**